# Experimental study and analytical model for the pore structure of epoxy latex-modified mortar

**DOI:** 10.1038/s41598-022-09836-z

**Published:** 2022-04-06

**Authors:** Pengfei Li, Wei Lu, Xuehui An, Li Zhou, Xun Han, Sanlin Du, Chengzhi Wang

**Affiliations:** 1 Department of Harbor, Waterway, and Coastal Engineering, Chongqing Jiaotong University, Chongqing, 400074 China; 2 State Key Laboratory of Hydroscience and Engineering, Tsinghua University, Beijing, 100084 China; 3 Geotechnical Engineering Department, Nanjing Hydraulic Research Institute, Nanjing, 210024 China; 4 Huaneng Tibet Hydropower Safety Engineering Technology Research Center, China Huaneng Group Co., Ltd., Beijing, 100031 China

**Keywords:** Composites, Mechanical properties

## Abstract

Concrete repair and rehabilitation prolong the effective service lives of structures and are important topics in the building field worldwide. Epoxy latex-modified cementitious materials have shown promise for a number of applications in building and construction, but the mix design processes remain arbitrary because their pore structures are not well understood. Porosity and pore size distributions are pore structure parameters that have direct effects on the mechanical properties and durability of concrete. In this paper, mercury intrusion porosimetry (MIP) was used to analyze the porosities and pore size distributions of epoxy latex-modified mortars. The effects of the polymer-to-cement ratio on the pore structures of epoxy latex-modified mortars were investigated. Mortars with polymer-to-cement ratios of 0%, 5%, 10%, 15%, and 20% were cured for 7, 28, 60, and 90 days in this study. Images of specimen microstructures were obtained by scanning electron microscopy (SEM), which showed that increases in the amount of epoxy latex added caused the proportion of micropores in the mortar to decrease, while the proportion of macropores and gel pores increased. The pore size distribution of epoxy latex-modified mortar was described with a composite logarithmic model. Relationships between the pore size distribution and the polymer-to-cement ratio and the curing age were obtained. The method described herein might be sufficiently accurate and convenient to evaluate or predict the pore size distribution of an epoxy latex-modified mortar, i.e., by determining the statistical distribution and analyzing the probability. The process for design of the polymer concrete mix ratio will be facilitated by methods that accurately describe the structure of the epoxy latex-modified mortar.

## Introduction

The pore structure is one of the most significant characteristics of cementitious materials, and it has major impacts on both mechanical properties^1–4^ and durability^5–7^. The pore structures of cementitious materials determine important properties such as strength^3,8^, permeability^9,10^, and shrinkage^11,12^. Cement-based materials with the same total porosity may also exhibit different properties. Therefore, it is important to investigate the porosity size distributions of cementitious materials to explain their properties.

Pore sizes, arrangements, and connections in cement-based materials are random and include gel pores (the interlayer pores in cement hydration produce calcium-silicate-hydrate gels), capillary pores, and air voids^13^. The pore distributions of cementitious materials are extremely complex because of the wide range and random distribution of pore sizes^14^. MIP is commonly used to investigate the pore structures of cementitious materials and is also one of the most widely used methods. The total porosity, pore size distribution, and density of a cementitious material can be obtained by MIP. The pores in cementitious materials are formed during hydration in the cement, and they mainly comprise two types of pores^1,15^: (a) gel pores, with sizes ranging from 0.5 to 10 nm, are mainly related to the cement hydration process and do not have a major impact on the strength of cementitious materials but have greater effects on the shrinkage and creep of the material; (b) capillary pores with sizes distributed primarily from 10 to 10,000 nm are strongly associated with the strength of a cementitious material, which is generally determined by the water-to-cement ratio of the material.

With the rapid developments occurring in the field of construction materials, synthetic polymer latexes, such as epoxy resin latex in cementitious material systems, have been used in many projects^16–19^. Polymer-modified cement mortar (PMM) is commonly used to enhance the physical properties and durabilities of structures because the reticulated film structure in the PMM can improve the pore structure^20–23^. Ordinary mortars have relatively more pores^24^. However, PMM fills the pores created during hydration of the cement by forming a polymer film in the mortar system^25^. As a result, PMMs based on various synthetic polymeric latexes are already being applied in the construction industry. Generally, the porosity and pore size distribution of the PMM system need to be considered when investigating the mechanical properties and durability of the material^26^. The physical properties and durability of ordinary cementitious materials are affected by pore structure parameters such as porosity and pore size distribution. However, the pore structure parameters of PMM materials may affect the mechanical properties and the durability of the material more than any other characteristic^27^. Therefore, the strengths, durabilities, and permeabilities of PMMs are strongly impacted by their pore structure parameters, such as porosities and pore size distributions^28,29^.

The microstructures of PMMs reported in the literature are mainly described by porosity and pore structure distribution. Then, the relationships between pore structure parameters and macroscopic properties are established to guide the application of PMMs in practical engineering. However, there are few accurate simulations of the pore structures of PMMs. The use of mathematical models to accurately describe the pore structure distribution has important implications for studies of PMM microstructures. Additionally, accurate descriptions of the pore structure and predictions of void structure parameters are important to promote the design of PMM mixtures. In view of the above, the pore structure parameters of PMMs obtained through MIP experiments, such as porosity and pore size distribution, can be used to investigate the relationships between polymer addition and pore structure distributions of cement mortars. This paper investigates the effects of different polymer-cement ratios and curing ages on the pore size structures of epoxy latex-modified cement mortars. MIP was used to obtain the pore structure changes of the specimens. The pore size distributions of epoxy latex-modified mortars were simulated with a compound log-normal distribution model. The model results were also compared with the test results of this study.

## Materials and methods

### Materials

**Figure 1:**
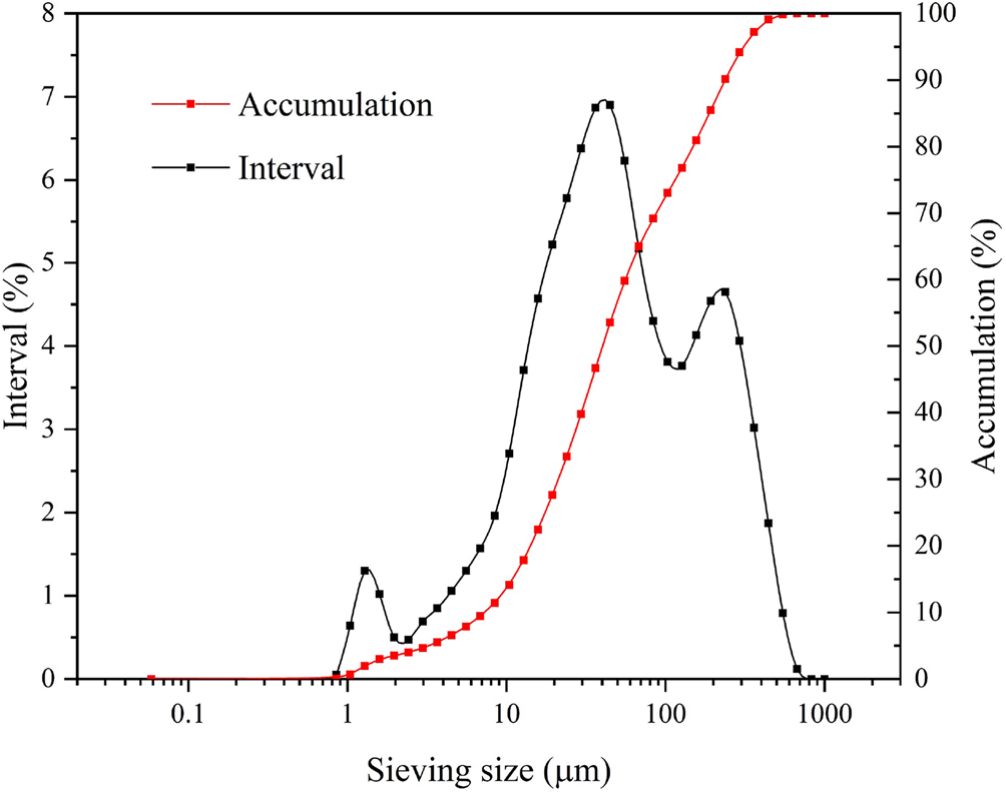
Cement particle size distribution curves.

**Figure 2:**
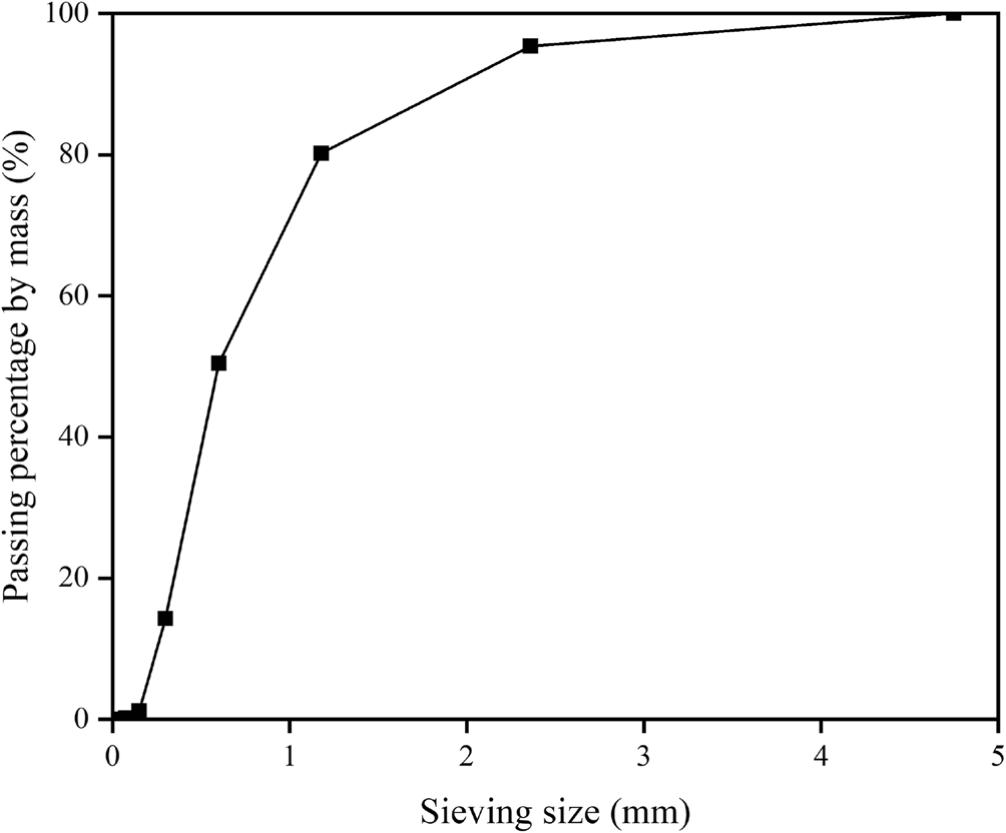
Particle size distribution curve for the quartz sand used in this investigation.

Ordinary Portland cement (PO 42.5) was used in this investigation, and it had a relative density of 3080 kg/m^3^. The specific surface area was 0.129 m^2^/g, as measured using a Bettersize 2000 (DL) laser particle-size analyzer. The cement particle-size distribution is shown in Fig. 1. The chemical composition of the cement determined by X-ray fluorescence spectrometry is shown in Table 1. The fine aggregate in this study was well-graded manufactured quartz sand. It had a specific gravity of 2700 kg/m^3^. The particle size distribution of the quartz sand was in the range 0.075–4.75 mm and is shown in Fig. 2.

**Table 1:** Chemical composition of the cement.

Chemical component	CaO	SiO_2_	Fe_2_O_3_	Al_2_O_3_	SO_3_	MgO	K_2_O	TiO_2_	BaO	SrO	Na_2_O
Content (wt.%)	69.42	14.7	4.21	3.67	3.2	1.55	1.42	0.597	0.489	0.265	0.184

**Figure 3:**
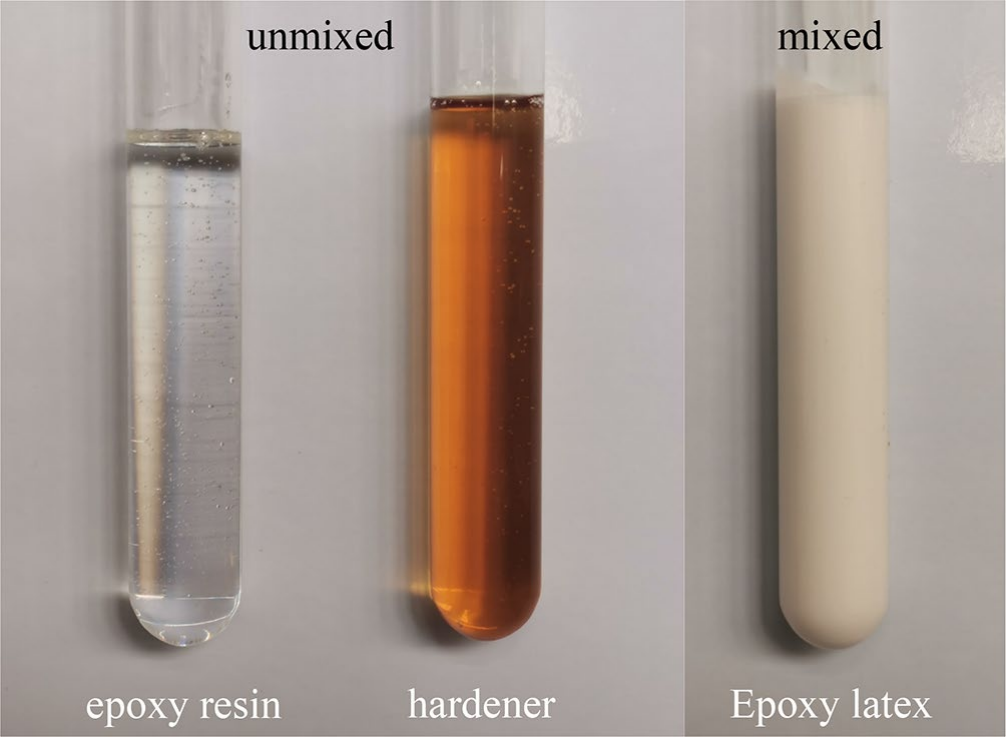
Images of epoxy latex materials before and after mixing.

Epoxy latex was prepared by emulsifying a bisphenol A-based epoxy resin in water with an emulsifier and an added hardener. As shown in Fig. 3, the epoxy latex used in this investigation was mainly composed of three parts: (a) bisphenol A-based epoxy resin, (b) water, and (c) hardener, with a solids content of 50%, which mainly consisted of a curing agent and emulsifier. The epoxy latex in this investigation had a density of 1.00–1.04 g/cm^3^ and a total solids content of 50 ± 1%.

### Specimen preparation

Previous studies showed that polymer-to-cement ratios of 5–15% were optimal for preparation of epoxy latex-modified mortars^20,27^. In our previous research^30,31^, we found that the compressive strengths of epoxy-modified mortars tended to decrease and the flexural strengths of epoxy-modified mortars tended to increase with increasing epoxy latex content. Therefore, epoxy resin-to-cement ratios of 0, 5%, 10%, 15%, and 20% were chosen to investigate the effects of the various contents of epoxy latex. The volumetric water-to-cement ratios of all mortar specimens were kept at 1.10. The cement-to-sand volume ratio was maintained at 1.00 for all specimens. Referring to the provisions of JC/T 986-2018^32^, all mortars were adjusted to exhibit a flowability of 200–210 mm by adding superplasticizers. The superplasticizer used has been applied in many previous studies^33–38^. Table 2 shows the mixing ratios for the materials in the epoxy latex-modified mortar. Furthermore, the *P/C* of the modified mortar indicates the dry epoxy resin-to-cement ratio of mass. The prepared epoxy emulsion-modified mortar was cast in square molds with dimensions of 70.7 mm. All specimens were cured at 100% RH and 20 °C for 1 day and then demolded. Then, the specimens were cured in a standard maintenance (60 ± 5% RH, 20 ± 1 °C) chamber for 7, 14, 28, or 90 days.

**Table 2:** Mix proportions for epoxy latex-modified mortars (kg/m^3^).

Mix label	*Vw*/*Vc*	*vs./Vc*	*P/C* (%)	C	S	W	ER	H
Control	1.10	1.00	0	832.70	1199.80	281.50	0.00	0
Latex 1			5				41.60	31.20
Latex 2			10				83.20	62.40
Latex 3			15				124.80	96.60
Latex 4			20				166.40	124.80

C, S, W, ER, and H are the quantities of cement, quartz sand, water, dry epoxy resin, and dry hardener, respectively.

### Experimental tests

#### Mercury intrusion porosimetry (MIP)

The MIP test is one of the most widespread methods used to investigate pore size distributions of cementitious materials. It provides accessible information, such as total pore volume, density, and pore size distribution for the cementitious material. MIP is very simple in principle. It is based on the physical principle that the applied pressure determines the extent of mercury intrusion into a porous medium. In the MIP experiment, the prepared sample is put into a chamber filled with mercury, and then the pressure applied to the mercury is steadily raised. With increasing pressure applied to the mercury, the volume of mercury pressed into the pores of the specimen also increases. To fill a pore with a diameter *d* with a nonwetting fluid, a pressure P that is inversely proportional to the diameter of that pore must be applied^39^. The Washburn equation, as shown below, can be utilized to express the association between applied pressure and pore size^40^:

1 $$D = \frac{ - 4\gamma \cos \varphi }{P}$$

where *P* = the absolute pressure applied; *φ* = the contact angle of the sample and mercury (132° used here); *γ* = the surface tension of mercury; and *D* = the diameter of the intruded cylinder.

MIP tests were performed herein on a Micromeritics Poresizer AutoPore IV 9500 instrument with a maximum intrusion pressure of 61,000 psi (420 MPa). The pores of two different specimens were tested, and average values were calculated for the test results. If the results of the two tests differed by more than 5%, it may have been due to experimental error, and a third specimen was tested.

#### Scanning electron microscopy (SEM)

SEM was conducted to obtain a clearer perspective on the microscopic effects of epoxy latex on cement mortar. The samples were immersed in absolute ethanol for 24 h to terminate hydration of cement and when the mortar specimens reached the ages of 7, 14, 28, or 90 days of maintenance. After drying the samples, SEM was conducted directly.

## Results and discussion

### Pore size distribution

**Figure 4:**
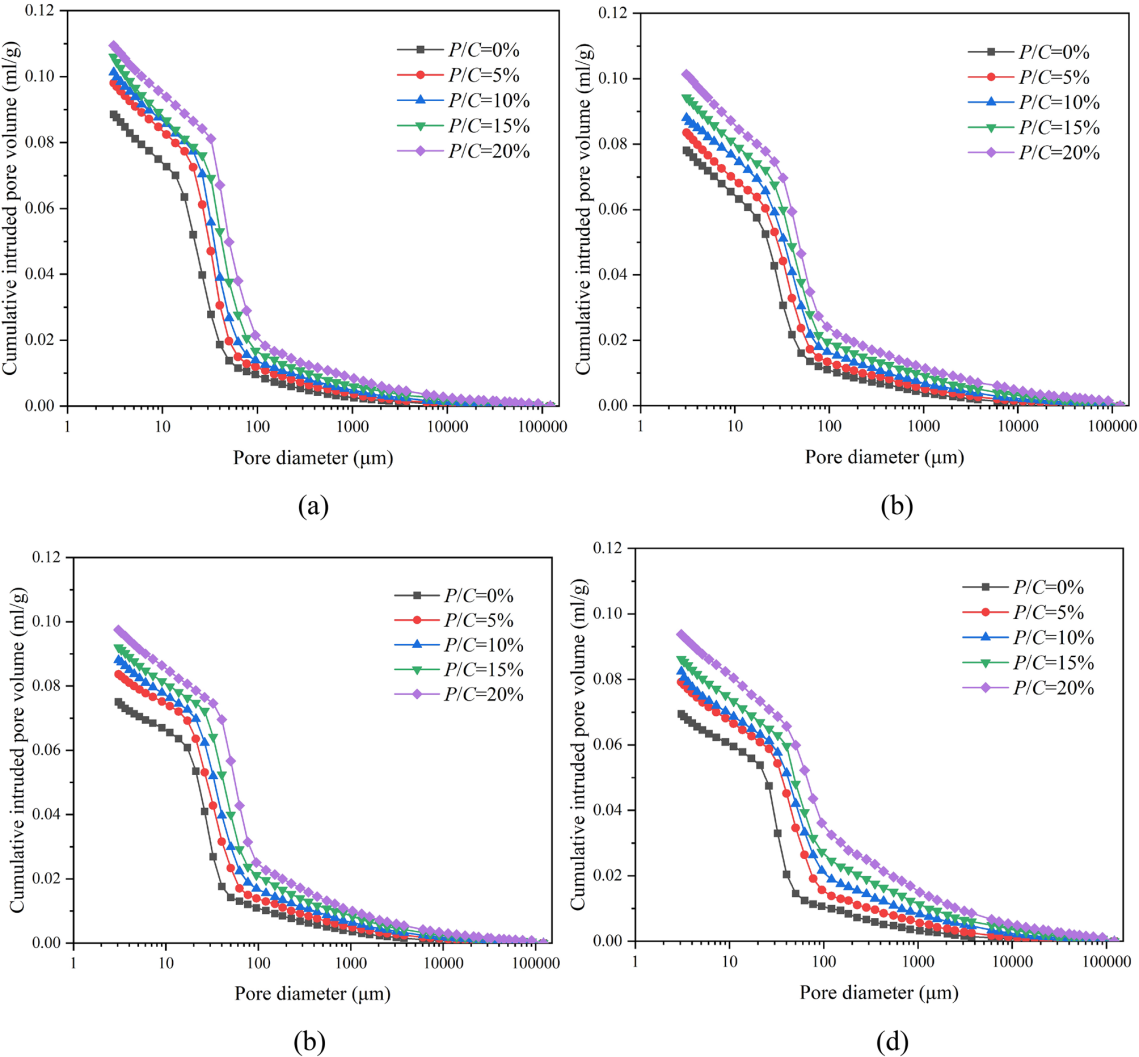
Relationship between the cumulative pore size distribution and *P/C* ratio of epoxy latex-modified mortar at different ages, (**a**) 7, (**b**) 14, (**c**) 28, and (**d**) 90 days.

**Figure 5:**
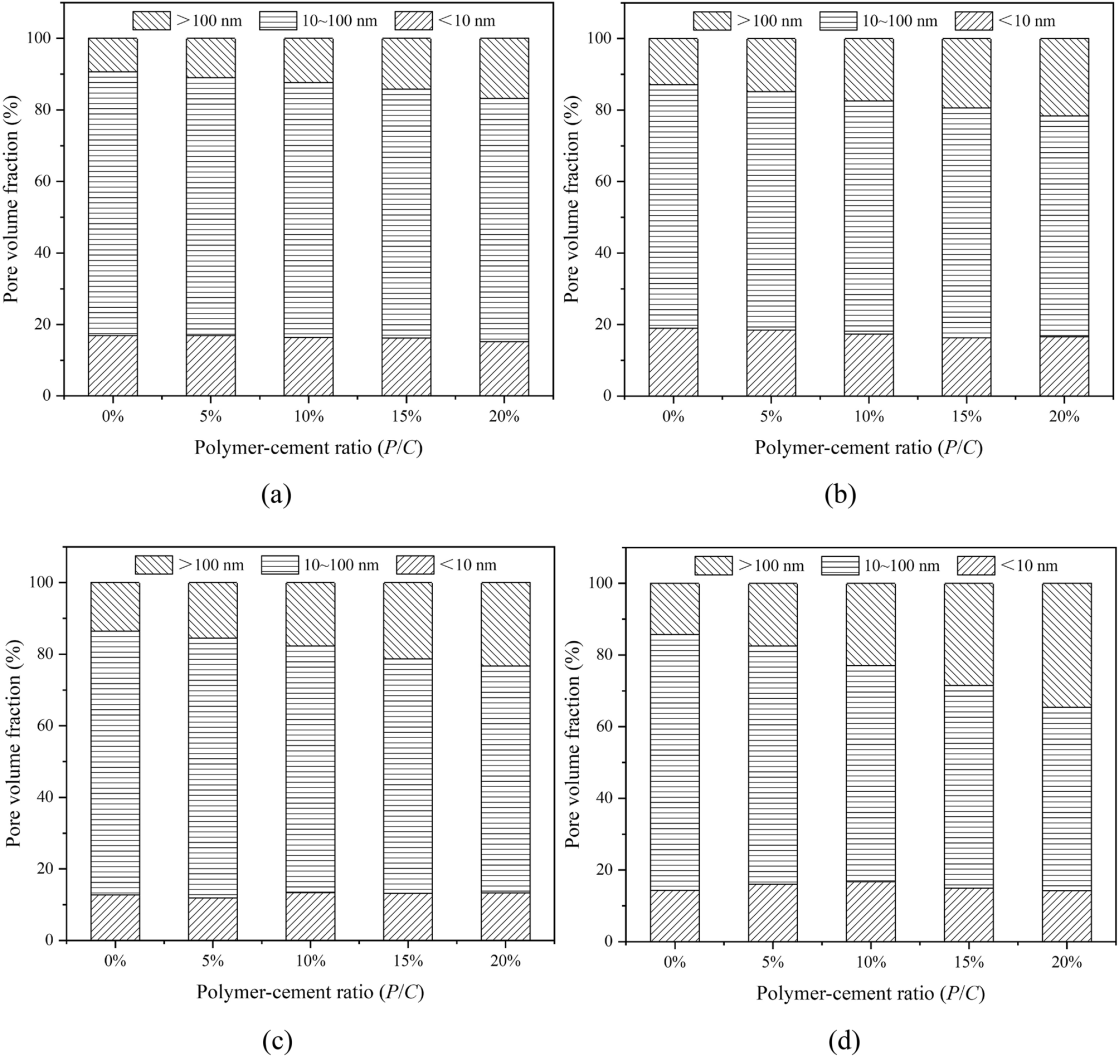
Pore volume distribution of epoxy latex-modified mortar with pore classifications at (**a**) 7, (**b**) 14, (**c**) 28, and (**d**) 90 days.

The pore structures of epoxy latex-modified mortars were obtained by MIP after 7, 14, 28, and 90 days of curing. Figure 4 shows cumulative pore diameter distribution curves for modified mortar specimens with different *P/C* ratios. As the *P/C* ratio increased, the pore size distribution curves of the specimens gradually shifted upward and to the right, and all of them were higher than that of the control mortar. This indicated that admixture of the epoxy latex led to an increase in the total pore space of the mortar. Similar results were reported in the literature^30,41^; polymer latexes introduced large pores into cement mortars, which increased the total porosities of PMMs. To better show the relationship between the *P/C* ratio and pore size distribution, the pore size distributions of the specimens were separated into three size ranges: gel pores (D < 10 nm), micropores (10–100 nm), and macropores (D > 100 nm)^39^. Figure 5 shows the classification of pore sizes. The pore sizes shown in the figure were mainly micropores and macropores. For epoxy latex-modified mortar, as the *P/C* ratio increased, the pore size increased substantially at all curing ages. For macropores, all samples had higher pore percentages with increasing *P/C* ratios, which is consistent with the results reported for epoxy cement mortars^27^, and the proportion of pores with sizes greater than 75 nm increased as the polymer-cement ratio increased. The micropore range has always had the largest proportion for all cases. Gel pore sizes of all epoxy latex-modified mortars increased with a smaller trend. A mixture containing epoxy latex has been shown to have a remarkable impact on the pore structure of epoxy latex-modified mortar during the curing process^26^.

### Compound log-normal distribution for pore size distributions

According to previous studies^42^, the pore size distribution determined by MIP can be simulated with a mixture of log-normal or composite log-normal distributions. It was found that the pore distributions of cement paste and mortar were well simulated by the composite log-normal distribution.

When a variable *x* (0 < *x* < ∞) meets the log-normal distribution, the function *y* = ln*x* is normally distributed. The probability density function is shown as follows:

2 $$p\left( x \right) = \frac{1}{{\sqrt {2\pi \sigma^{2} x} }}\exp \left[ { - \frac{1}{2}\left( {\frac{\ln x - \mu }{2}} \right)^{2} } \right]$$

where *x* is a variation, *μ* is defined as the location parameter, and *σ* is the shape parameter. In the log-normal distribution model, there are several relevant characteristics, as follows:

3 $${\text{mean }} = \exp \left( {\mu + 0.5\sigma^{2} } \right)$$

4 $${\text{median }} = \exp \left( \mu \right)$$

5 $${\text{mode}} = \exp \left( {\mu - \sigma^{2} } \right)$$

6 $${\text{coefficient of variation }} = \exp \left( {\sigma^{2} } \right) - 1$$

7 $${\text{variance }} = {\text{ mean}}^{{2}} \left[ {\exp \left( {\sigma^{2} } \right) - 1} \right]$$

Generally, the hydration process for cement can be considered a process of dividing the voids of the cement system or the spaces between particles in the cement system. In the early stages of hydration, there are large voids in the cement paste system, i.e., voids between cement particles. As curing progresses, the cement particles are connected by cement hydration products. Therefore, the large voids in the cement paste system are divided into smaller pores. This suggests a physical basis for the log-normal model of pore size distribution in cement systems^42^.

The probability density function of the composite log-normal distributions model is shown as follows:

8 $$p\left( x \right) = f_{1} p\left( {x,\mu_{1} ,\sigma_{1} } \right) + f_{2} p\left( {x,\mu_{2} ,\sigma_{2} } \right) + f_{3} p\left( {x,\mu_{3} ,\sigma_{3} } \right)$$

9 $$\sum\limits_{i = 1}^{3} {f_{i} = 1}$$

where *μ*_*i*_ = the location parameter, *σ*_*i*_ = the shape parameter, *f*_*i*_ = the weight coefficient, and *p*(*x*, *μ*_*i*_, *σ*_*i*_) = the *i*th log-normal subdistribution. Research showed that if two or more log-normal distributions explained an attribute of a system, it is likely that there were multiple distinct response processes occurring in that system^43^. This may indicate that the different size ranges of pores in cementitious materials had different sources and generation mechanisms.

**Figure 6:**
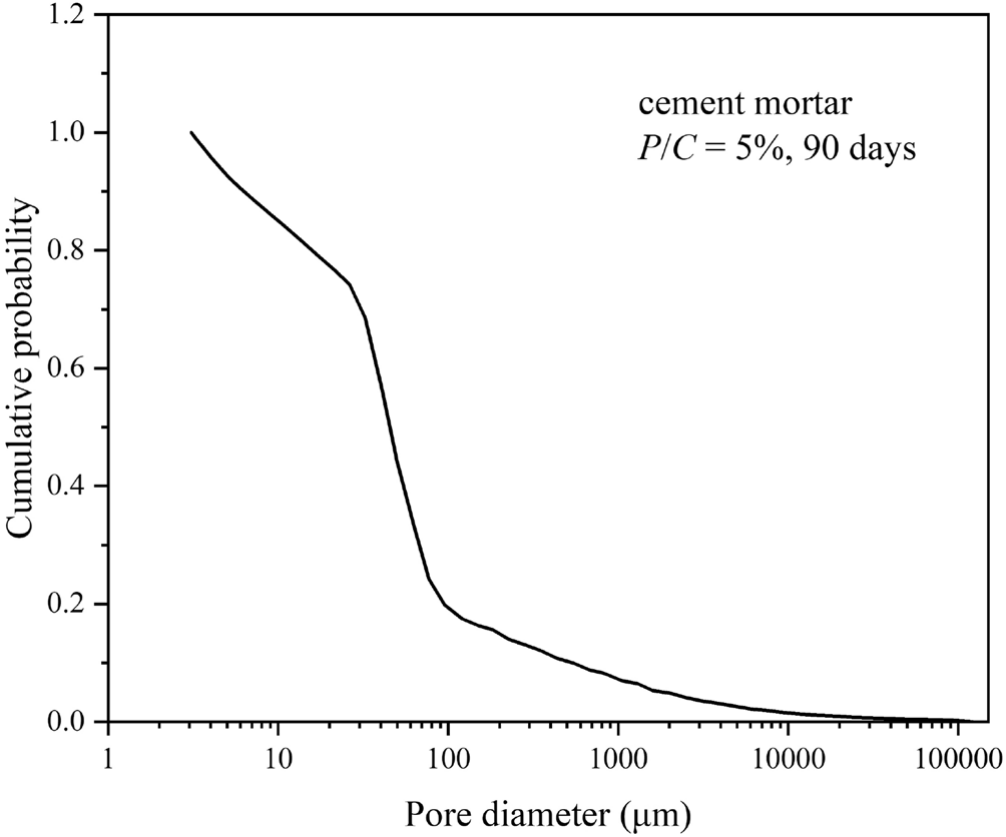
Cumulative pore volumes in epoxy latex-modified mortars hydrated in *P/C* = 5% for 90 days.

The method for modeling pore size distributions in this study was obtained from a group of cumulative probability data. The relationship between *P*(*x*) and *x* is shown in Fig. 6. In this investigation, *x* = the pore diameter and *P*(*x*) = the ratio of the volume of the cumulative pore to the total pore determined by MIP tests. In general, the distribution model and the initial values of the parameters are frequently determined by graphical analysis. This graphical approach first requires the logarithmic transformation of *x* to obtain ln*x*. Then, the standard normal distribution table is used to determine the quantiles of N(0,1) determined by *P*(*x*). For instance, if the cumulative percentage is 80%, the corresponding quantile is 0.86. With ln*x* as the vertical axis and the quantiles of N(0,1) as the horizontal axis, if the curve of ln*x* versus quantiles of N(0,1) is linear in shape, then *x* can be considered to obey a single log-normal distribution.

**Figure 7:**
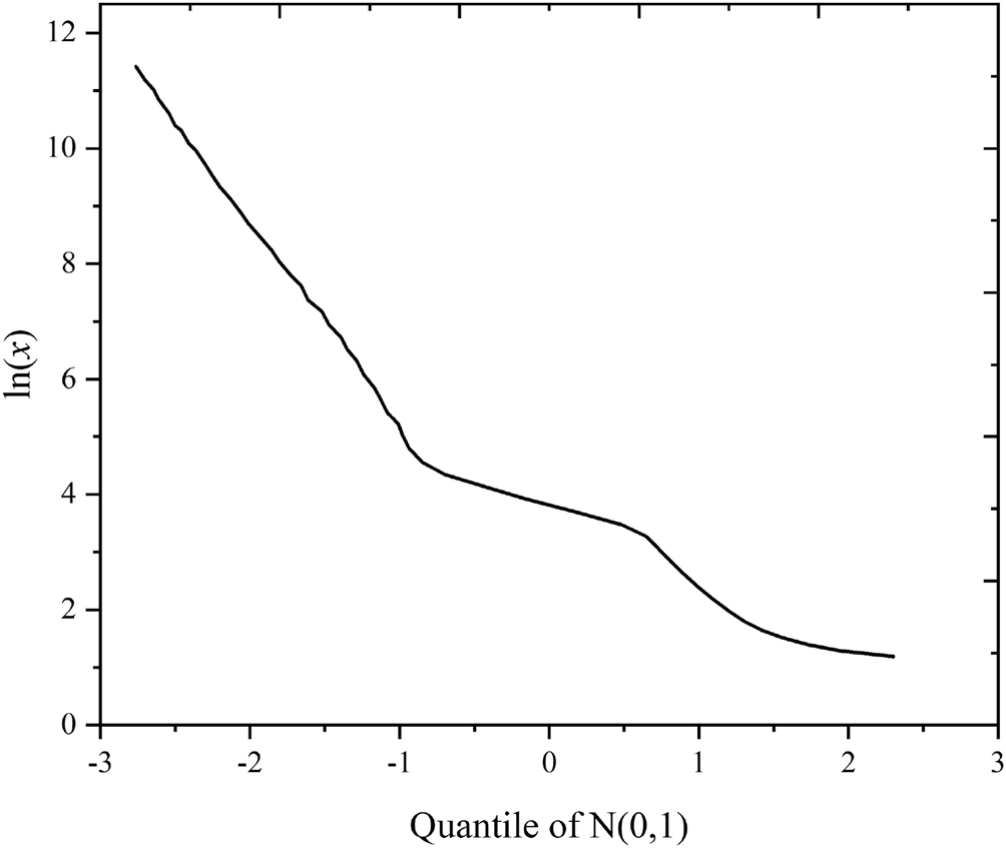
Plot of ln*x* versus the quartiles of N(0,1) obtained based on the data in Fig. 6.

**Figure 8:**
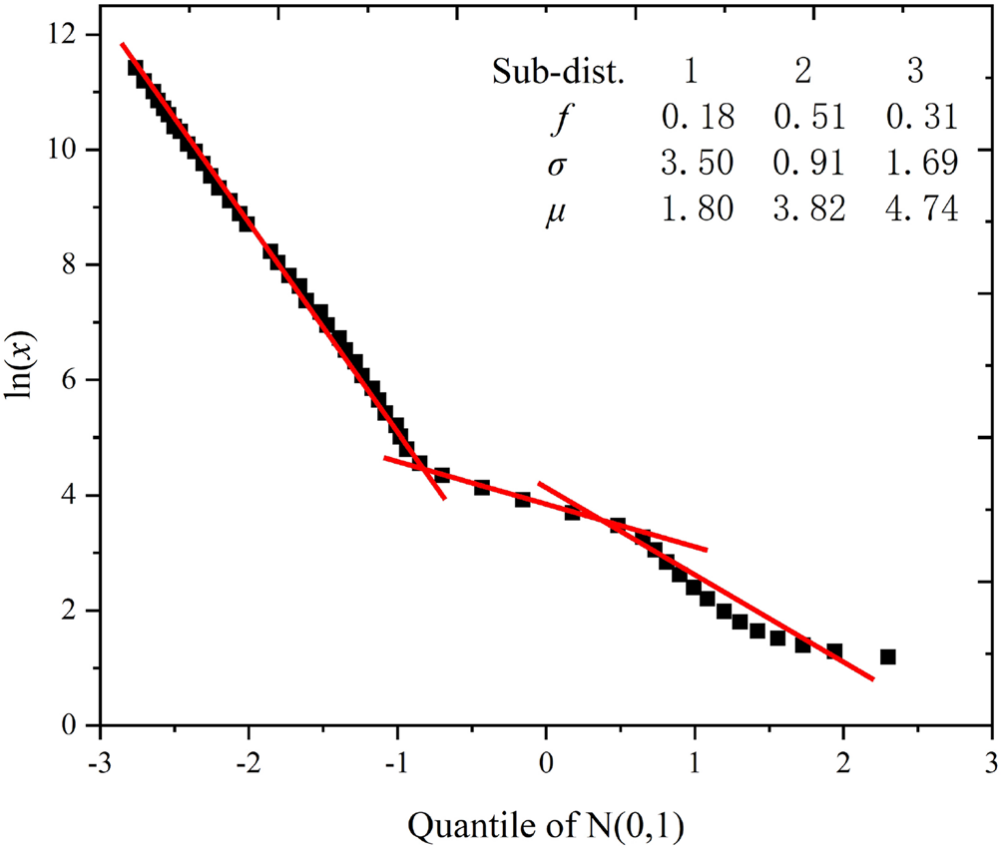
Initial prediction parameter acquisition method for the composite log-normal distribution.

The curves for plots of ln*x* versus the quantiles calculated from the MIP data for epoxy latex-modified mortar specimens are shown in Fig. 7. Notably, the curves comprised three straight line segments. As shown in Fig. 8, the slope and intercept of the straight line represented the location (*μ*_*i*_) and shape (*σ*_*i*_) parameters of the corresponding subdistribution, respectively. The horizontal axis at the intersection points of adjacent line segments determined the weighting factor. As shown in Fig. 8, the first segment of the line intersected the second segment at quantile − 0.91, and the corresponding cumulative probability was identified as 0.18 by consulting the standard normal distribution table. Thus, *f*_1_ = 0.18.

The test results for epoxy latex-modified mortars of different ages were compared with the results from the distribution fitted using the *χ*^*2*^ fitting technique. It is well known that the χ^2^ test is one of the important indicators used to evaluate whether the fitted data are good or not. This method for evaluating the goodness of fit is as follows:

10 $$\chi_{0}^{2} = \sum\limits_{i = 1}^{k} {\frac{{\left( {O_{i} - E_{i} } \right)^{2} }}{{E_{i} }}}$$

where χ^2^ = the test statistic, *k* = the number of bins, *O*_*i*_ = the observed frequencies, and *E*_*i*_ = the expected frequencies. The most appropriate distribution is obtained by adjusting the initially predicted distribution parameters of the model to obtain the smallest possible test statistic. Subsequently, the critical values of $\chi_{\alpha ,k - \beta - 1}^{2}$ with *k* − *β* − *1* degrees of freedom are calculated. The α-error is considered to be 0.05. If $\chi_{0}^{2} > \chi_{\alpha ,k - \beta - 1}^{2}$, the hypothesis that the pore size data for the epoxy latex-modified mortar followed a compound log-normal distribution should be rejected.

**Figure 9:**
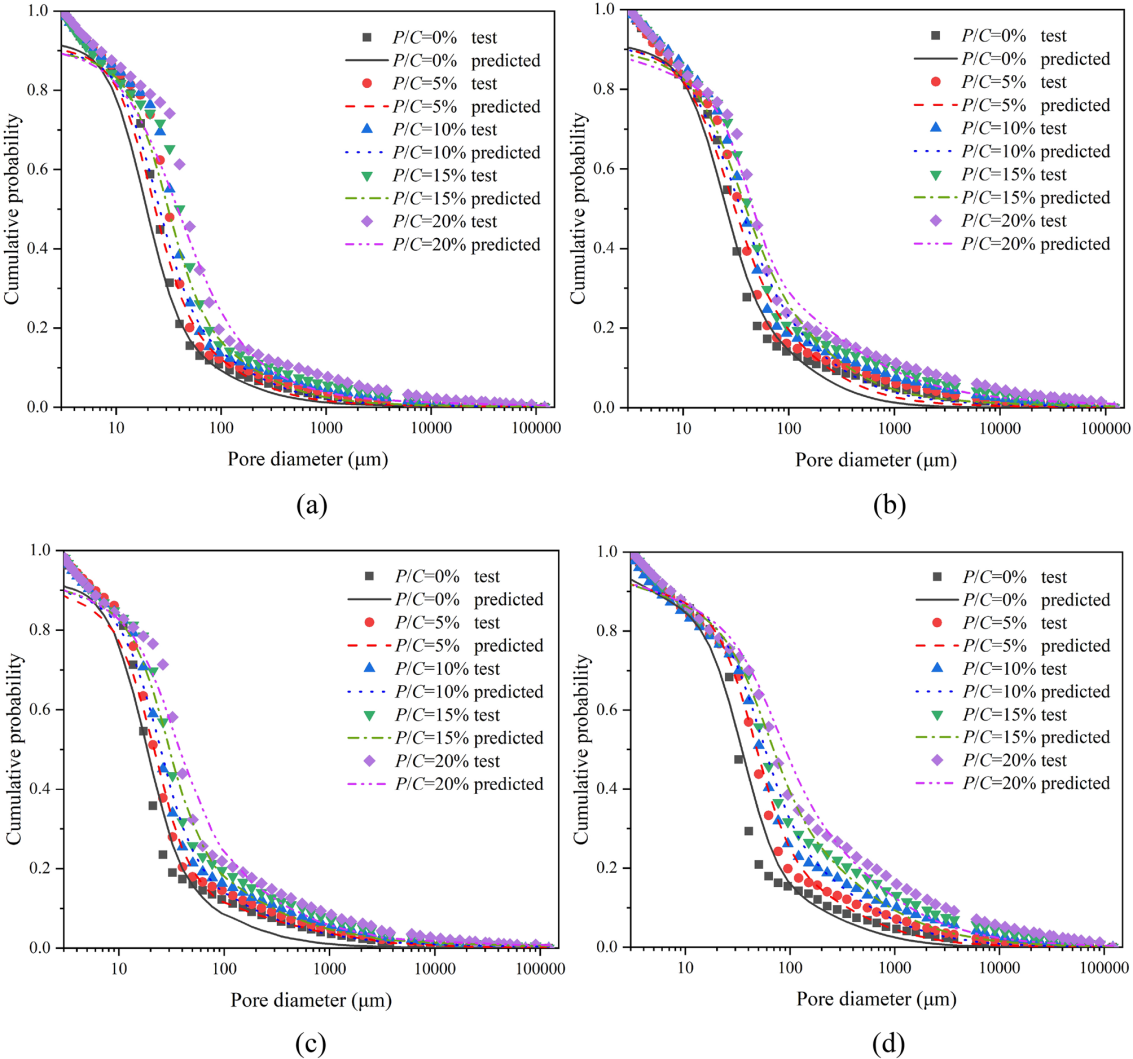
Relationships between predicted and tested cumulative pore size distributions of epoxy latex-modified mortars at (**a**) 7, (**b**) 14, (**c**) 28, and (**d**) 90 days.

Table 3 and Fig. 9 indicate the differences in pore size distribution between the model and experimental results. For all mortars, the inequalities $\chi_{0}^{2} < \chi_{\alpha ,k - \beta - 1}^{2}$ were satisfied, and their correlation coefficients were close to 1. As shown in Fig. 9, the experimental data for the pore size distribution of epoxy latex modified mortar were in excellent agreement with the model curves obtained. Therefore, the composite log-normal distribution used in this study predicted the cumulative pore size distribution curves of epoxy latex-modified mortars with different *P/C* ratios effectively.

**Table 3:** Compound log-normal distribution parameters for the pore size distributions of epoxy latex-modified mortars.

Age (days)	*P/C* (%)	*f*_1_	*σ*_1_	*μ*_1_	*f*_2_	*σ*_2_	*μ*_2_	*f*_3_	*σ*_3_	*μ*_3_	*R*^2^	$\chi_{0}^{2}$	$\chi_{\alpha ,k - \beta - 1}^{2}$
7	0	0.13	4.27	− 0.08	0.64	0.72	2.88	0.23	1.40	3.91	0.99	0.45	72.15
	5	0.14	4.20	0.11	0.59	0.70	3.03	0.27	1.45	4.01	0.99	0.60	72.15
	10	0.18	4.18	0.23	0.54	0.68	3.12	0.28	1.42	4.14	0.99	0.75	72.15
	15	0.21	4.24	0.33	0.49	0.75	3.26	0.30	1.50	4.22	0.99	1.00	72.15
	20	0.24	4.26	0.50	0.45	0.76	3.37	0.31	1.45	4.30	0.99	0.75	72.15
14	0	0.17	4.21	0.28	0.50	0.76	3.15	0.33	1.42	4.17	0.99	3.71	72.15
	5	0.18	4.14	0.68	0.46	0.79	3.29	0.36	1.46	4.29	0.99	1.13	72.15
	10	0.19	4.20	0.90	0.44	0.85	3.40	0.37	1.43	4.40	0.99	2.00	72.15
	15	0.22	4.24	1.12	0.39	0.81	3.50	0.39	1.45	4.49	0.99	1.15	72.15
	20	0.24	4.29	1.37	0.35	0.83	3.73	0.41	1.47	4.66	0.99	1.58	72.15
28	0	0.15	3.84	0.64	0.64	0.70	3.33	0.21	1.60	4.33	0.99	1.30	72.15
	5	0.18	3.85	0.97	0.61	0.75	3.53	0.21	1.62	4.44	0.99	1.26	72.15
	10	0.20	3.90	1.31	0.57	0.76	3.62	0.23	1.60	4.60	0.99	0.33	72.15
	15	0.21	3.84	1.82	0.52	0.73	3.79	0.27	1.65	4.75	0.99	0.27	72.15
	20	0.22	3.91	2.03	0.43	0.76	3.96	0.35	1.63	4.83	0.99	0.30	72.15
90	0	0.14	3.53	1.18	0.59	0.96	3.55	0.27	1.76	4.65	0.99	2.81	72.15
	5	0.18	3.50	1.80	0.51	0.91	3.82	0.31	1.69	4.74	0.99	0.59	72.15
	10	0.23	3.58	2.51	0.43	0.94	3.96	0.34	1.77	4.96	0.99	1.78	72.15
	15	0.29	3.53	3.12	0.34	0.95	4.09	0.37	1.74	5.06	0.99	1.73	72.15
	20	0.33	3.55	3.67	0.27	0.93	4.29	0.40	1.73	5.27	0.99	0.80	72.15

### Pore parameters of epoxy latex-modified mortars

The composite model with three log-normal distributions may provide considerable information on the origins and formation methods for pores of various sizes. Physically, the size distribution of macropore pores (D > 100 nm) can be described by the first subdistribution. The size distribution of gel pores (D < 10 nm) can be described by the third subdistribution. The middle subdistribution represents micropore pores (10 nm ≤ D < 100 nm). Table 3 showed that *f*_*2*_ decreased continuously with increasing *P/C* ratio. In this case, the micropores in the modified mortar system decreased as the *P/C* ratio increased, which is consistent with the results of previous studies^30^ showing that the proportion of pores with sizes ranging from 50 to 100 nm tended to increase with increasing polymer dosage. As the *P/C* ratios increased from 5 to 20%, the proportion of macropore pores increased, and *f*_3_ increased to a small degree; this can be explained by the fact that some of the micropores were filled by the hardened epoxy. A mass of aggregated epoxy resin particles adsorbed cement particles, which led to an increase in macropores. This result is consistent with literature^44^ indicating that the number of pores (with size ranges 0.1 μm to 1 μm and 10–200 μm) increased with increasing incorporation of polymer. Adsorption of the epoxy emulsion on cement particles hindered the hydration process in the mortar system, which resulted in an increased number of gel pores^30,44^.

**Figure 10:**
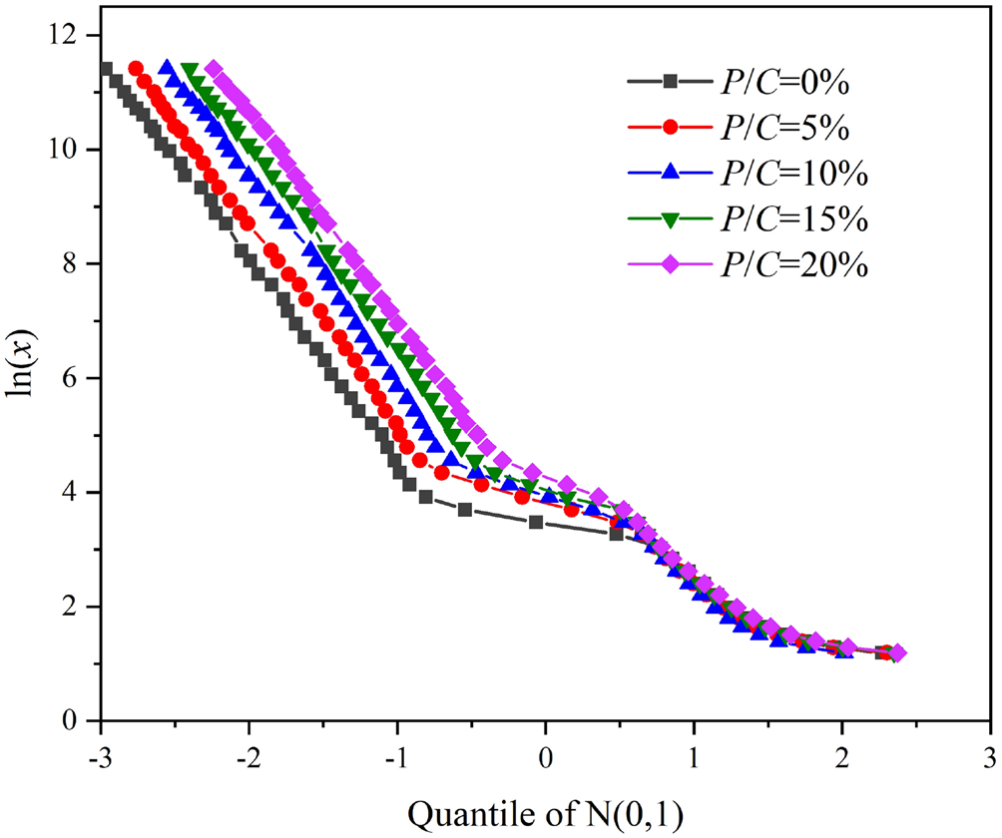
Q-ln plots for epoxy latex-modified mortar samples cured for 90 days.

Figure 10 shows Q-ln plots for epoxy latex-modified mortar samples cured for 90 days. Initial estimates for the slopes of the three fairly linear segments were designated *σ*_1_, *σ*_2_, and *σ*_3_. Initial estimates for the intercepts of the three segments were designated *μ*_1_, *μ*_2_, and *μ*_3_, respectively. The two deflection points were the initial estimates for *f*_1_ and *f*_2_. It is obvious what the weighing factors *f*_1_, *f*_2_, and (1- *f*_1_- *f*_2_) mean. Therefore, it is necessary to investigate the relationships among location parameters, shape parameters and *P/C* ratios of the epoxy latex-modified mortar pore structures. As shown in Fig. 10, the linear segments at the ends of the curves were almost parallel to each other. This indicates that the shape parameters *σ*_*1*_, *σ*_*2*_, and *σ*_*3*_ of the log-normal pore size distributions in the modified mortar systems remained essentially constant as the *P/C* rate increased. Therefore, in the epoxy latex-modified mortar system, the shape parameter in the composite log-normal distribution may be related to other factors. Thus, it is necessary to investigate the relationship between the position parameter and the *P/C* ratio.

### Prediction of the location parameter of epoxy latex-modified mortar

**Figure 11:**
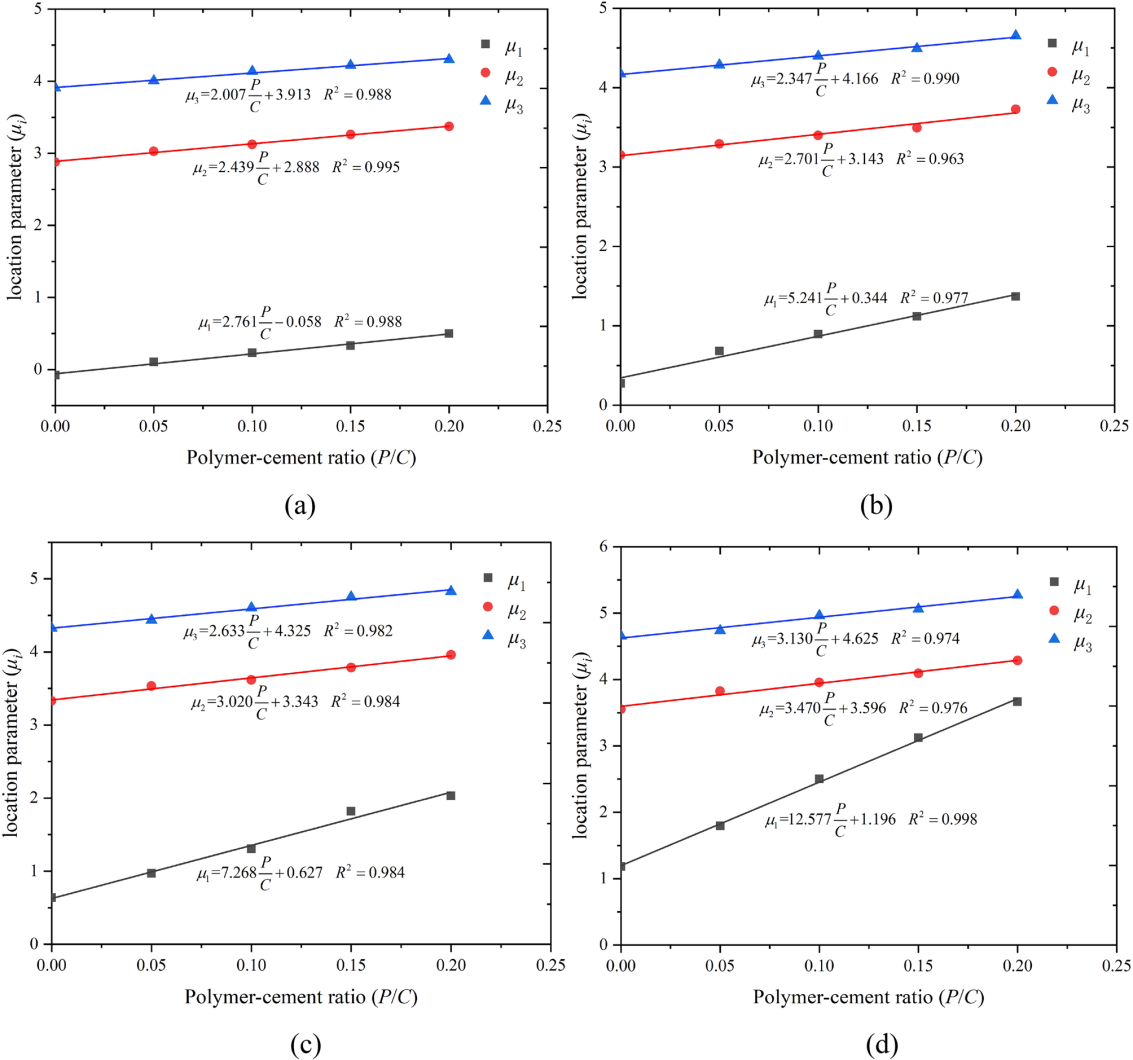
Relationships between location parameters (*μ*_*i*_) and polymer-to-cement ratios (*P/C*); (**a**) 7, (**b**) 14, (**c**) 28, and (**d**) 90 days.

The location parameter (*μ*_*i*_) relative to the polymer-to-cement ratio (*P/C*) was studied first. It was found that *μ*_*1*_, *μ*_*2*_, and *μ*_*3*_ may be related to the epoxy latex. For the same curing age, it was shown that variations in the location parameters with respect to the *P/C* ratio were best represented by the relationship $\mu_{i} = a\left( {{P / C}} \right) + b$. A plot of the location parameter against the *P/C* ratio is shown in Fig. 11. The regression equation and the corresponding correlation coefficients are shown in Fig. 11. As shown, the location parameter increased with increases in the *P/C* ratio. This indicates that the pore structure of epoxy latex-modified mortar evolved toward large pores as the *P/C* ratio increased, which further supports previous findings^45^ that increases in the polymer latex percentage increased the total porosity for a constant water-to-cement ratio. The relationships between the location parameters and the *P/C* ratios were linear with correlation coefficients as high as *R*^*2*^ > 0.960.

**Figure 12:**
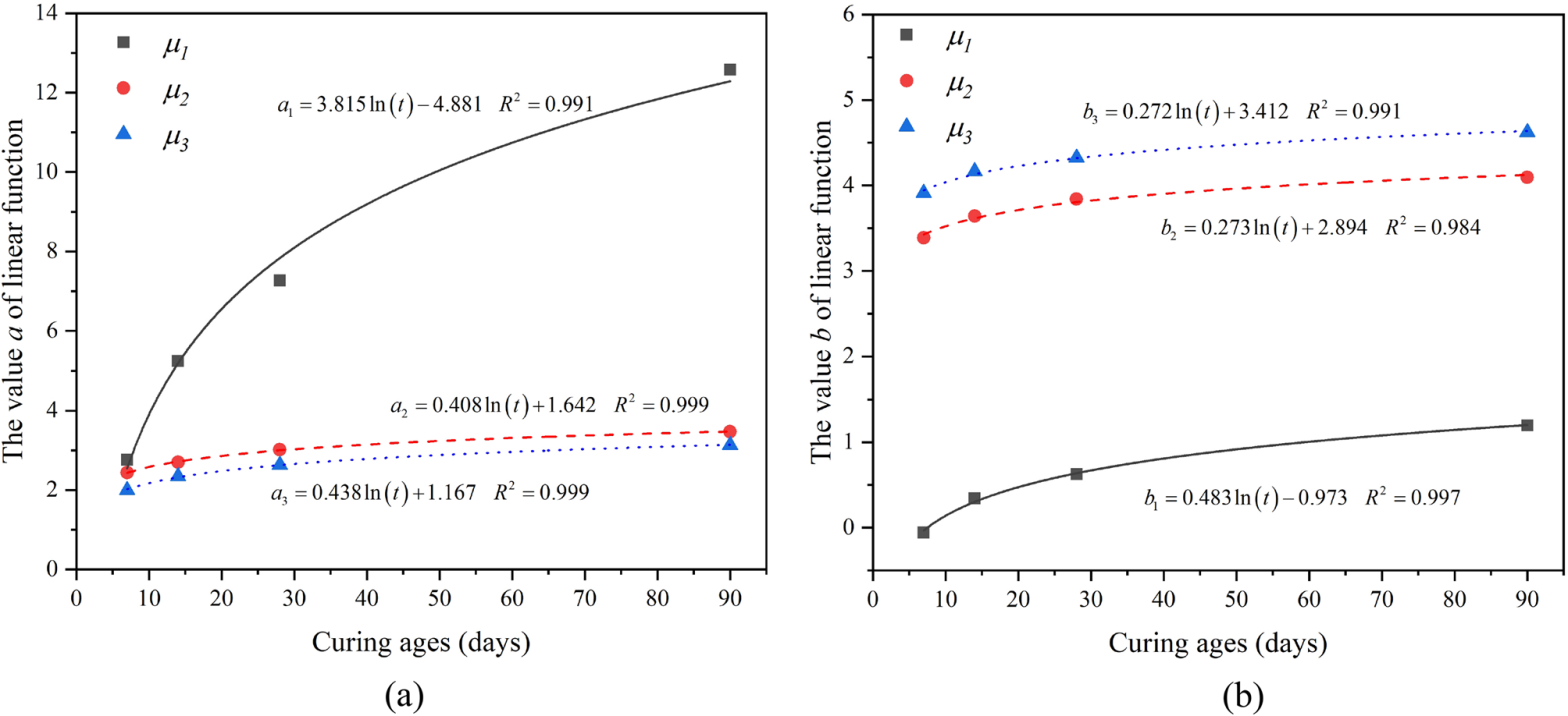
Relationships between the values *a* and *b* of a linear function and curing ages, (a) *a* and (b) *b*.

Furthermore, the values *a* and *b* for the linear plots of location versus the curing age are plotted in Fig. 12. It has been observed that the variations of a and b with age t are best expressed as a relationship of the following form; $a,b = c\ln \left( t \right) + d$, where *t* is the curing age. The regression equation and the corresponding correlation coefficients are also shown in Fig. 12. It is interesting to note that the plots of values *a* and *b* of the linear function versus the curing age showed logarithmic relationships with correlation coefficients as high as *R*^*2*^ > 0.980.

As concluded above, Equations *a* and *b* were substituted into the equation $\mu_{i} = a\left( {{P/C}} \right) + b$. Accordingly, the relationships between location parameters (*μ*_*i*_) and polymer-to-cement ratios (*P/C*) and curing age (*t*) can be expressed as follows:

$$\begin{aligned} & \mu_{1} = \left( {3.815\ln t - 4.881} \right)\left( \frac{P}{C} \right) + 0.483\ln t - 0.973, \\ & \mu_{2} = \left( {0.408\ln t + 1.642} \right)\left( \frac{P}{C} \right) + 0.273\ln t + 2.894, \\ & \mu_{3} = \left( {0.438\ln t + 1.167} \right)\left( \frac{P}{C} \right) + 0.272\ln t + 3.412. \\ \end{aligned}$$

### Effect of epoxy latex on the microstructure of mortar

**Figure 13:**
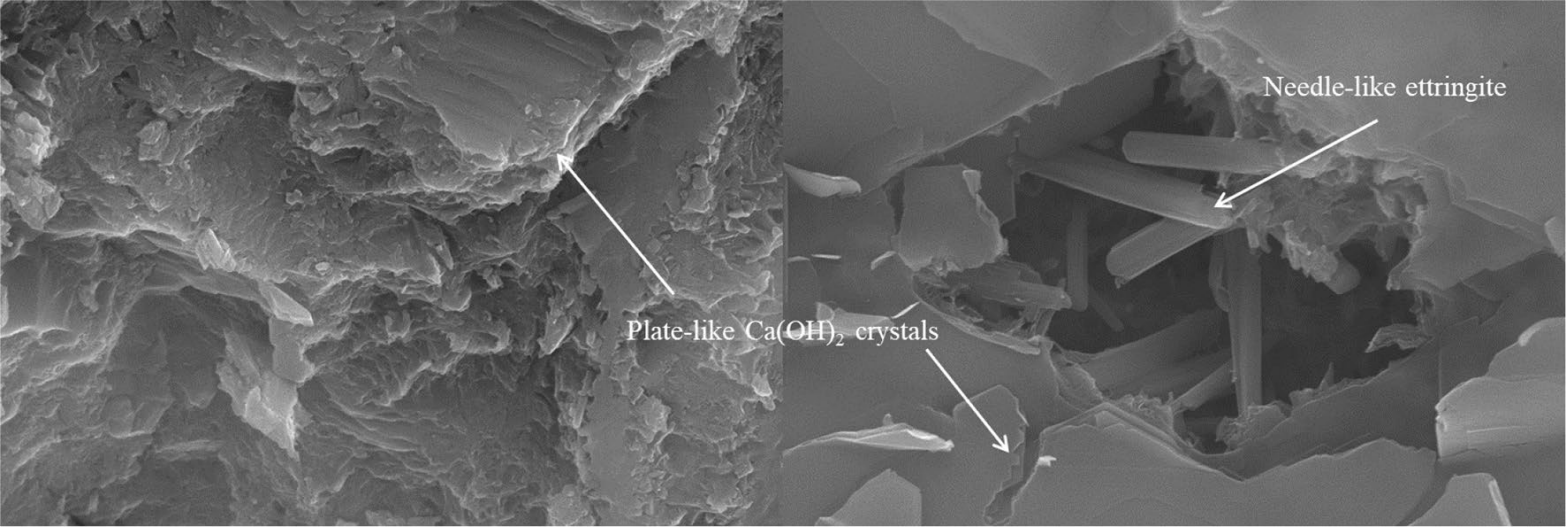
SEM images of ordinary cement mortar specimens cured for 28 days.

**Figure 14:**
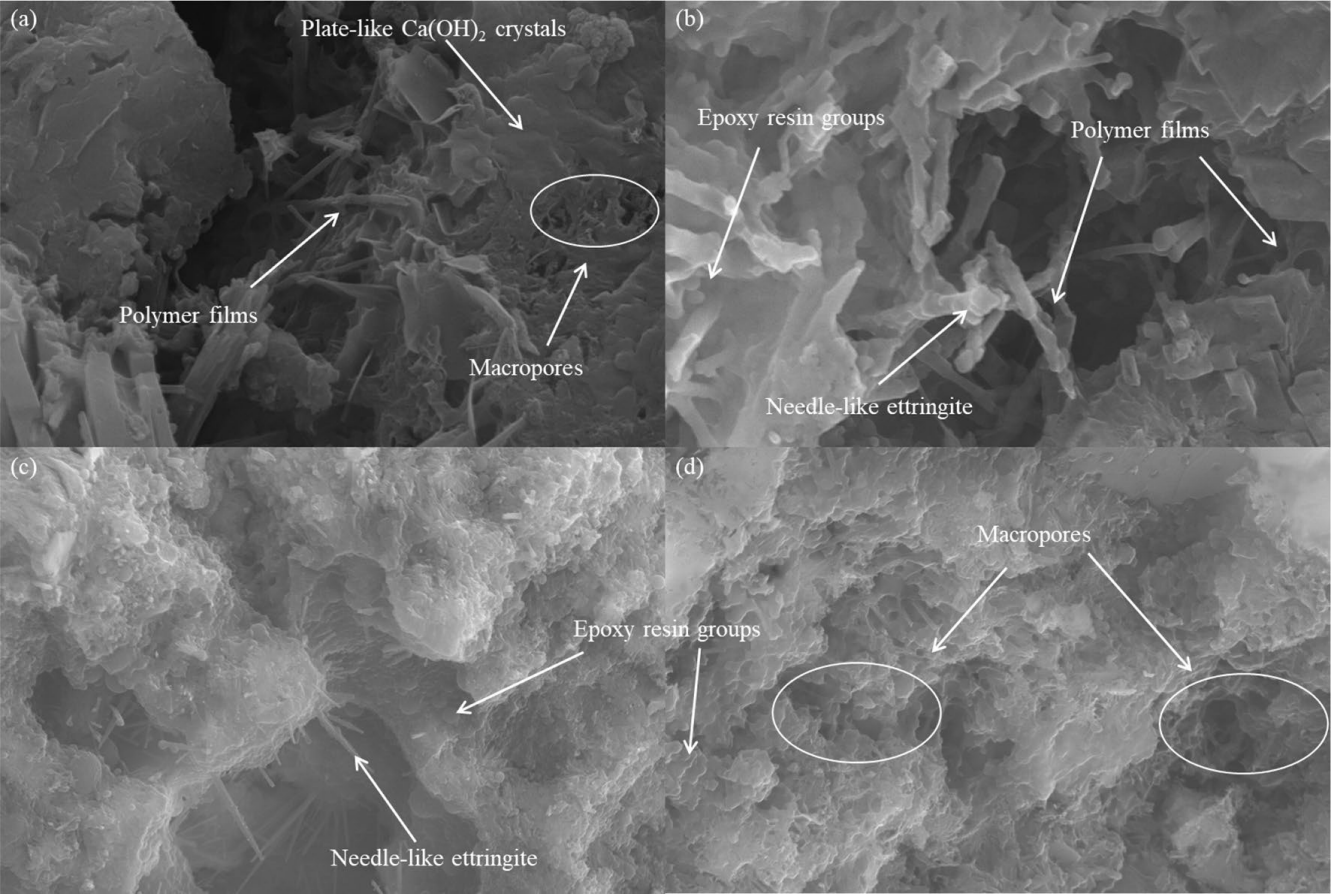
SEM images of modified mortar specimens containing epoxy latex cured for 28 days: (**a**) *P/C* = 5%, (**b**) *P/C* = 10%, (**c**) *P/C* = 15%, and (**d**) *P/C* = 20%.

The microstructures of an ordinary cement mortar and an epoxy latex-modified mortar are shown in Figs. 13 and 14, respectively. Figure 13 shows that a large number of plate-like and needle-like products were generated in the normal mortar. These needle-like ettringite products and plate-like Ca(OH)_2_ crystals were the main products of cement hydration, and these products interwove to form a dense structure, which contributed to improvements in the pore structures of cementitious materials^46^. Figure 14 shows that, as with the control mortar, Ca(OH)_2_ crystals and ettringite products were formed in the epoxy latex-modified mortar. However, polymer films were observed for the epoxy latex-modified cement mortar, and these films were beneficial to improving the pore structure of the cement mortar^21^. As seen from Fig. 14a, when 5% epoxy latex was added to the cement mortar, pores were observed on the surfaces of the plate-like Ca(OH)_2_ crystals. As shown in Fig. 14b, at a *P/C* ratio of 10%, round lumps were formed in the modified mortar. This may be due to adsorption of cement particles by epoxy aggregates. Figure 14c shows the microstructure of an epoxy latex-modified mortar with *P/C* = 15%. This showed that the round lumps formed by the epoxy aggregates completely covered the hydration products, which led to generation of more macropores. As shown in Fig. 14d, when the addition of epoxy latex was increased to 20%, a large number of macropores and round lumps were formed in the mortar system. More macropores were formed in the modified mortar system as the amount of epoxy latex added was increased, which was consistent with previous findings^27^.

## Conclusions

This paper investigated the effects of different polymer-to-cement ratios (*P/C*) and curing ages on the pore size structures of epoxy latex-modified cement mortars. The following conclusions can be drawn from this research:

1. With increasing *P/C* ratios, the proportion of micropores in epoxy latex-modified mortars tended to decrease, while the proportion of macropores tended to increase. The addition of epoxy latex hindered the hydration process of cement in the mortar system, which led to an increase in the proportion of gel pores.
2. The addition of epoxy latex was only associated with the location parameters. The shape parameter in the composite log-normal distribution was independent of epoxy doping.
3. With increases in the *P/C* ratio, the epoxy latex-modified mortar developed larger pores.
4. A composite logarithmic model was used to describe the pore size distribution of epoxy latex-modified mortar. The relationships between pore size distribution location parameters and the polymer-to-cement ratio and curing age were also obtained. The analytical methods proposed in this study can be applied in other studies of pore size distributions.
5. With increases in the amount of epoxy latex added, larger numbers of epoxy groups were formed in the modified mortar system, which led to the generation of more macropores.
6. From the above test results, polymer to cement ratios ranging from 10 to 15% were considered optimal for preparation of such epoxy latex cement mortars.
